# Artificial Intelligence in Cardiovascular Risk Prediction: An Up-to-Date Narrative Review on the Emerging Role of Lipid Profile-Based Models

**DOI:** 10.3390/jcm15156053

**Published:** 2026-08-04

**Authors:** Patrycja Piłat, Radosław Dutczak, Mariusz Gąsior, Przemysław Trzeciak

**Affiliations:** 1 3rd Department of Cardiology, Silesian Center for Heart Disease in Zabrze, Faculty of Medical Sciences in Zabrze, Medical University of Silesia in Katowice, 41-800 Zabrze, Poland; m.gasior@op.pl (M.G.); przemyslaw.t@wp.pl (P.T.); 2 Department of Physiology, Faculty of Medical Sciences in Katowice, Medical University of Silesia in Katowice, 40-752 Katowice, Poland

**Keywords:** artificial intelligence (AI), lipid profile, cardiovascular risk (CVR), machine learning, clinical practice

## Abstract

**Introduction**: Cardiovascular risk prediction remains challenging, particularly in patients with intermediate risk, mixed dyslipidemia, elevated lipoprotein(a), or variable lipid profiles. Conventional risk calculators may not fully capture nonlinear relationships among lipid, clinical, imaging, and longitudinal data. **Objectives**: This narrative review summarizes evidence on artificial intelligence (AI)-based cardiovascular risk assessment, focusing on lipid profile-based and multimodal models incorporating lipid-related variables. **Methods**: PubMed/MEDLINE, Scopus, and Google Scholar were searched for English-language articles published up to January 2026. Original studies, reviews, and relevant clinical guidelines addressing AI-based cardiovascular risk models, lipid-related predictors, and clinically applicable approaches were considered. **Results**: Lipid profile-based AI models may identify lipid phenotypes and lipid-related patterns associated with increased cardiovascular risk, while multimodal models have shown improved performance in selected datasets. However, the reviewed studies address heterogeneous tasks, including phenotype classification, cardiovascular event prediction, mortality prediction, patient trajectory modeling, and absolute risk estimation. Most evidence remains retrospective, with limited external validation, calibration assessment, and clinical utility data. **Conclusions**: AI-based models may support cardiovascular risk assessment, but routine implementation requires prospective validation, standardized evaluation, calibration, explainability, and clinical impact studies.

## 1. Introduction

Cardiovascular diseases remain a major challenge for contemporary preventive cardiology and continue to be among the leading causes of death worldwide [1]. Early identification of patients at high risk of cardiovascular events remains imperfect, despite significant advances in the diagnosis and treatment of acute coronary syndromes [2]. The probability of experiencing a cardiovascular event within a defined time horizon is referred to as cardiovascular risk (CVR) and is based on the combined effects of established risk factors. The lipid profile constitutes one of the fundamental components in the pathophysiology of atherosclerosis, and assessment of its parameters plays a key role in CVR stratification. The lipid profile constitutes one of the fundamental components in the pathophysiology of atherosclerosis, and assessment of its parameters plays a key role in cardiovascular risk (CVR) stratification. Importantly, several lipid parameters, including low-density lipoprotein cholesterol (LDL-C) and lipoprotein(a) (Lp(a)), have been shown to represent independent risk factors for cardiovascular disease, beyond traditional clinical predictors. At the same time, cardiovascular risk assessment also incorporates non-lipid clinical factors, including age, sex, blood pressure, and cigarette smoking, which together with lipid parameters form the basis of traditional risk stratification models [3].

The lipid profile comprises the assessment of key parameters of lipid metabolism, including total cholesterol (TC), low-density lipoprotein cholesterol (LDL-C), high-density lipoprotein cholesterol (HDL-C), triglycerides (TG), and, increasingly, lipoprotein(a) [Lp(a)] [4]. LDL-C is the principal atherogenic lipid fraction, and according to the guidelines of the European Society of Cardiology (ESC) and the European Atherosclerosis Society (EAS), its concentration represents the primary therapeutic target of lipid-lowering therapy [5].

Higher HDL-C levels have been associated with protective effects through antioxidant, anti-inflammatory, and antithrombotic mechanisms, whereas elevated concentrations of TG and Lp(a) are associated with an increased risk of cardiovascular events, independently of other established risk factors [6,7].

In current clinical practice, several tools are used for CVR assessment, such as the Systematic Coronary Risk Estimation 2 (SCORE2) and Systematic Coronary Risk Estimation 2-Older Persons (SCORE2-OP) models, which incorporate demographic variables (age, sex), clinical parameters (blood pressure, smoking), and biochemical variables (total cholesterol or LDL-C) [8].

Other tools are also available, such as the American Heart Association’s PREVENT™ calculator, which allows estimation of 10- and 30-year cardiovascular risk based on the lipid profile, renal function, and metabolic parameters [9]. Ten-year risk estimation for atherosclerotic cardiovascular disease (ASCVD) can also be performed using the ASCVD Risk Estimator Web App [10]. These models were developed based on analyses of existing population cohorts [8]. Risk calculators perform well in general populations; however, their accuracy is limited in patients with advanced metabolic disorders, such as mixed dyslipidemia or elevated Lp(a), which may lead to underestimation of actual cardiovascular risk [4,11]. These risk calculators are primarily intended for primary prevention and estimate the risk of a first cardiovascular event in individuals without established cardiovascular disease. In patients with established atherosclerotic cardiovascular disease, residual cardiovascular risk refers to the risk that remains after initial lifestyle modification and treatment of major risk factors [11]. The need for improved risk assessment therefore applies to both primary and secondary prevention settings, and the present review discusses lipid profile-based AI models within both clinical contexts. Several recent narrative and systematic reviews have highlighted the expanding role of artificial intelligence (AI) in preventive medicine and primary health care, particularly in the prevention of cardiovascular disease. AI-based approaches have been proposed to improve cardiovascular risk stratification, identify previously unrecognized high-risk individuals, facilitate opportunistic screening, support personalized preventive strategies, and assist clinicians through clinical decision support systems using routinely collected electronic health records, laboratory findings, and other clinical data. These applications are particularly relevant in primary care, where most cardiovascular prevention strategies are initiated and long-term cardiovascular risk management is delivered [12,13,14].

Despite these advances, recent reviews consistently emphasize that most AI models remain at the research stage and have not yet undergone adequate prospective or external validation. Limited model explainability, heterogeneous datasets, algorithmic bias, regulatory challenges, and difficulties integrating AI into routine clinical workflows remain major barriers to widespread implementation. Furthermore, although several recent reviews have summarized the role of AI in preventive cardiology and primary health care, comparatively little attention has been devoted specifically to AI models incorporating lipid biomarkers, despite dyslipidemia being one of the most important modifiable cardiovascular risk factors for cardiovascular disease. This gap provides the rationale for the present narrative review, which focuses on the emerging role of lipid profile-based AI models in contemporary cardiovascular risk prediction [12,13,14].

The development of analytical tools based on artificial intelligence (AI) offers opportunities to improve CVR assessment [15]. AI, and particularly machine learning (ML) methods, enable the analysis of multidimensional datasets, identification of nonlinear relationships, and modeling of complex interactions between biochemical and clinical variables [16].

The aim of this review was to evaluate the role of AI in cardiovascular risk prediction, with particular emphasis on models based on lipid profile parameters. In addition, multimodal AI models incorporating lipid biomarkers together with other clinical, laboratory, imaging, and electronic health record (EHR) data are discussed to place lipid-based approaches in the context of contemporary cardiovascular risk prediction.

## 2. Methods

This narrative review was conducted to summarize the current evidence regarding AI-based models for cardiovascular risk prediction, with particular emphasis on lipid profile-based approaches. Relevant literature was identified through searches of PubMed/MEDLINE, Scopus, and Google Scholar for articles published up to January 2026. The search strategy included combinations of the following keywords: artificial intelligence, machine learning, deep learning, cardiovascular risk, lipid profile, dyslipidemia, LDL cholesterol, lipoprotein(a), cardiovascular disease prediction, and risk stratification. Original research articles, systematic reviews, and relevant clinical guidelines published in English were considered. Priority was given to peer-reviewed studies directly addressing AI-based cardiovascular risk prediction, particularly those evaluating lipid-related predictors and clinically applicable predictive models. Articles were selected according to their relevance to the review topic, methodological quality, and potential clinical applicability. The principal studies and selected contextual examples of AI-based cardiovascular risk prediction and clinical implementation discussed in this review are summarized in Table 1.

Since the included studies differed in their modeling objectives and reported endpoints, we described each model according to its primary task or use case, distinguishing lipid phenotype classification, cardiovascular event prediction, mortality prediction, patient trajectory prediction, and absolute risk estimation where applicable.

## 3. AI-Based Modeling Approaches in Cardiovascular Risk Assessment

In recent years, numerous AI models have been developed to support the diagnosis, monitoring, and prediction of cardiovascular events [29]. Predictive models integrating imaging, laboratory, and clinical data have gained particular importance, as some models have shown higher predictive performance than traditional risk scores in selected datasets [30].

AI-based clinical tools rely on predictive models that must first be trained and validated. In models for CVR prediction, the most applied approaches involve supervised learning methods, in which models are trained on datasets with known clinical outcomes and input variables [31]. Models should ideally be evaluated using independent validation datasets, although many published models rely primarily on internal validation [32]. The effectiveness of these models depends on high-quality, well-standardized data, with laboratory parameters, including the lipid profile, being an important part of this dataset [33].

Key methods used to train predictive models used in assessing CVR:

- Random Forests (RF) and Gradient Boosting Machines (GBM)—ensemble methods capable of identifying complex, nonlinear relationships among variables, from laboratory parameters to imaging results, with relatively interpretable feature importance [34].
- Bidirectional Long Short-Term Memory networks (BiLSTM)—recurrent neural networks capable of incorporating the temporal history of clinical events; BiLSTM networks may improve risk prediction in settings where temporal patterns are clinically relevant (e.g., progressive metabolic disease or lipid variability) [35].
- Deep learning models—neural networks suitable for multidimensional data, including imaging (ECG, echocardiography, CT). These models can be designed to provide automated predictions in complex datasets, although clinical utility depends on validation, interpretability, and workflow integration [36].
- Support Vector Machines (SVM)—machine learning classifiers that learn to assign patients to appropriate risk categories based on a limited number of features, such as lipid parameters. SVM classifiers may support risk categorization using limited feature sets, although their performance depends on feature quality and validation strategy [37].

**Figure 1:**
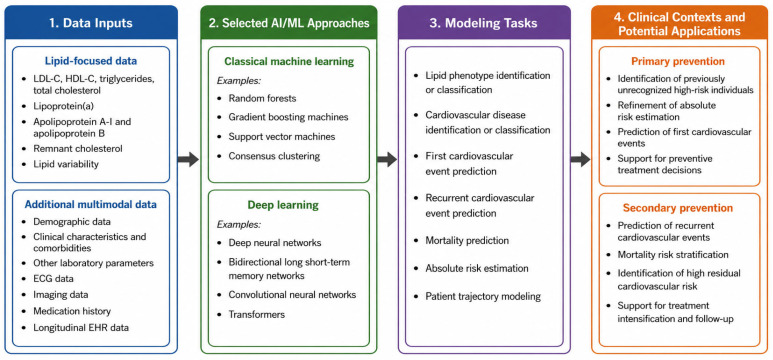
From lipid-focused and multimodal data to AI-based cardiovascular applications.

Methods described above are not limited to multimodal datasets; they can also be applied to selected subsets of routinely available clinical data. Consequently, AI-based models discussed in this field include approaches based primarily on lipid profile parameters as well as multimodal models integrating lipid-related variables with additional clinical, imaging, or longitudinal data. Figure 1 illustrates how lipid-related and additional multimodal data may be analyzed using selected AI approaches to address distinct modeling tasks and support potential applications in primary and secondary prevention.

## 4. AI-Based Models Using Lipid Profile Parameters

Several studies have examined the use of lipid-related variables in AI-based cardiovascular risk assessment, ranging from lipid phenotype classification to cardiovascular event prediction, mortality prediction, and absolute risk estimation, demonstrating the potential of lipid parameters as independent predictors.

In a study by Xue et al., AI methods were applied to analyze lipid profiles of 1355 patients with STEMI who underwent percutaneous coronary intervention between 2015 and 2018 [17]. Seven lipid parameters (TC, TG, HDL-C, LDL-C, apoA1, apoB, and Lp(a)) were used to classify patients into three lipid phenotypes. Group 1 was characterized by higher Lp(a) levels and reduced HDL-C and apoA1 concentrations. Group 2 exhibited higher HDL-C and apoA1 levels together with lower TG, TC, LDL-C, and apoB concentrations, while group 3 showed elevated TG, TC, LDL-C, and apoB levels accompanied by lower Lp(a) concentrations. Therefore, this study should be interpreted primarily as an example of AI-based lipid phenotype classification, with subsequent assessment of associations between identified phenotypes and long-term clinical outcomes.

Patients in Group 1 showed significantly worse long-term outcomes (median follow-up 904 days), including higher mortality and rehospitalization rates, compared with the other groups. Multivariable analysis confirmed that Group 1 had the highest risk of adverse events, including death.

In a larger retrospective cohort of 23,548 patients with hyperlipidemia, Du et al. evaluated four different machine learning models for predicting cardiovascular events over a three-year follow-up [18]. The analysis suggested the prognostic value of lipid biomarkers, including lipid variability (temporal fluctuations in lipid levels) and remnant cholesterol (cholesterol content in atherogenic lipoproteins other than LDL-C and HDL-C). In this cohort, the best-performing machine learning model showed higher predictive performance than classical logistic regression and selected traditional risk scores; however, these findings were derived from a retrospective dataset and require external validation before clinical application. Omitting lipid variability or remnant cholesterol from the analysis significantly reduced predictive accuracy.

Sadiq et al. applied AI to assess ASCVD risk associated with dyslipidemia in 112 pregnant women, using the atherogenic index of plasma (AIP), defined as the logarithm of the TG-to-HDL-C ratio [19]. Higher TG levels and lower HDL-C were most strongly associated with increased risk. Among the models tested, Random Forest achieved the highest predictive performance (R^2^ = 0.95, a measure of the proportion of variance in the outcome explained by the model), surpassing GBM (R^2^ = 0.923) and classical decision trees (R^2^ = 0.76). This study illustrates that AI models based on lipid parameters may help identify individuals with higher predicted risk, although larger prospective validation studies are needed.

These studies suggest that lipid-related variables can contribute to different AI-based modeling tasks, including lipid phenotype classification, risk-related classification, and cardiovascular event prediction. However, it should be noted that these models were developed and tested retrospectively and are not yet deployed as clinical tools; further prospective validation would be required for clinical implementation.

## 5. Multimodal AI Models Incorporating Lipid Profile Parameters

Contemporary AI-based cardiovascular risk assessment models increasingly integrate multiple data sources, including lipid parameters, demographic characteristics, clinical variables, imaging data, and longitudinal electronic health records [38]. In a UK Biobank-based study, Islam et al. applied a range of machine learning algorithms to primary care and imaging data from over 500,000 participants. These models integrated demographic data (age, sex), clinical variables (e.g., diabetes, hypertension), laboratory measures (LDL, HDL), and imaging data (echocardiography). The models were used for ASCVD-related identification or classification, with the authors reporting an accuracy of up to 96%. The analysis was based on UK Biobank data collected between 2006 and 2010, with additional follow-up visits conducted in 2014. However, because the study has not yet undergone peer review and focused on disease identification or classification rather than prospective cardiovascular event prediction or absolute risk estimation, its findings should be considered preliminary. Nevertheless, it provides a contextual example of a multimodal approach in which lipid parameters were incorporated as part of a broader clinical dataset [20].

In a preprint by Bagheri et al., a multimodal BiLSTM-based model was developed by integrating unstructured electronic health record data, including radiology reports and clinical notes, with structured variables such as lipid levels and blood pressure. The model was evaluated in patients with vascular disease or high cardiovascular risk. BiLSTM architectures can capture temporal dependencies in longitudinal clinical data by accounting for the sequence and evolution of clinical events over time. In the evaluated dataset, the authors reported higher predictive performance than selected traditional risk calculators [21]. However, these findings should be interpreted with caution, as the study is currently available only as a preprint and has not undergone formal peer review. Its generalizability requires confirmation through independent external validation, while its clinical utility should be assessed prospectively.

In the study by Kwon et al., a deep learning model was developed to predict 12-month all-cause mortality in patients hospitalized with STEMI or NSTEMI [22]. The model included several dozen variables—demographic, clinical, and laboratory data (including lipid parameters), infarct type, and administered therapy. In this AMI cohort, the model achieved high discrimination for 12-month mortality (AUC = 0.88), showing higher performance than GRACE and TIMI risk scores. Importantly, it maintained strong performance even in challenging subgroups, such as patients with reduced left ventricular ejection fraction.

In the study by Ward et al., electronic health records of 262,923 adult patients from Northern California were analyzed [23]. At the start of the observation period, these patients had no prior diagnosed cardiovascular disease and were not on statin therapy. In addition to variables used in traditional risk calculators (such as age, sex, race, total cholesterol, HDL-C, systolic blood pressure, hypertension treatment, smoking, and diabetes) machine learning models incorporated over 1100 auxiliary variables from electronic records, including prescribed medications, ICD-9/10 diagnoses, educational and income indicators, number of medical visits, and laboratory test results. The GBM model achieved high discrimination for five-year ASCVD risk prediction, distinguishing patients who subsequently developed ASCVD from those who remained free of diagnosed cardiovascular disease during follow-up. Its performance exceeded that of traditional risk calculators under the evaluated conditions.

Recent advances in AI-based CVR prediction have increasingly highlighted the importance of longitudinal modeling approaches that incorporate temporal changes in clinical parameters rather than relying solely on isolated measurements obtained at a single time point. CVR is a dynamic process influenced not only by the current lipid profile, but also by changes in lipid levels over time, treatment exposure, progression of metabolic disorders, worsening renal function, and recurrent cardiovascular events [24,25].

In this context, transformer-based architectures and survival-oriented deep learning models have emerged as promising tools for the analysis of longitudinal electronic health records (EHRs) and patient trajectories. In contrast to traditional approaches based primarily on static clinical variables, these models can integrate sequential clinical information and temporal dependencies, which may support more dynamic and individualized CVR assessment [24,25,26]. Rao et al. recently developed the transformer-based TRisk model for absolute cardiovascular risk estimation and refined selection of individuals who may benefit from preventive cardiovascular interventions. The model was reported to improve identification of individuals who may benefit from preventive cardiovascular interventions in the evaluated cohorts [24]. Similarly, transformer-based survival models have been evaluated for mortality prediction in patients with heart failure by incorporating longitudinal multimorbidity patterns and time-dependent clinical changes [25]. Furthermore, recently developed transformer-based frameworks for patient trajectory prediction, such as ETHOS, have been proposed as a framework for predicting future health trajectories from longitudinal EHR data, supporting further investigation of foundation models for longitudinal risk prediction and precision cardiovascular medicine [26].

Collectively, these findings suggest that longitudinal and transformer-based AI models may represent an important future direction in precision cardiovascular medicine and AI-assisted CVR stratification [24,25,26]. These longitudinal AI frameworks may also facilitate continuous remote monitoring of patients with cardiovascular disease, including individuals following cardiac surgery, by supporting clinical decision-making through integration of longitudinal electronic health records and multimodal clinical data [19,21,22,23].

## 6. Clinical Implementation and Interpretability of AI-Based Cardiovascular Models

The potential clinical applications of AI-based cardiovascular models differ between primary and secondary prevention. In primary prevention, these models may refine absolute risk estimation, identify individuals whose risk is underestimated by conventional calculators, and support decisions regarding the initiation or intensification of preventive interventions. In secondary prevention, they may support the prediction of recurrent cardiovascular events or mortality, identify patients with high residual risk and inform the intensity of therapy and follow-up. The substantial and continuously expanding number of AI-enabled medical devices authorized by regulatory agencies, including the US Food and Drug Administration, illustrates the growing regulatory recognition of AI-based technologies in medicine. Cardiovascular applications are clearly represented within this broader landscape [39]. However, the vast majority of these systems are designed to support diagnostics or automate measurements, rather than serve as independent prognostic risk estimators. Consequently, although AI models based on the lipid profile are being actively studied, their application in predicting cardiovascular risk remains at the testing stage and they are not yet implemented in clinical practice.

A notable example illustrating the potential of such tools in the future is the AIRE (AI-ECG Risk Estimation) model [27]. While it is based solely on ECG analysis, it is an example of an algorithm capable of assessing cardiovascular risk (CVR). The tool was developed to estimate 10-year risk of myocardial infarction, heart failure, arrhythmias, and all-cause mortality, and in a study including over 1.6 million patients, it showed higher performance than traditional risk calculators, particularly in individuals with intermediate risk. For clinicians, such tools may support more precise identification of patients who may benefit from intensified preventive strategies. Starting in 2025, AIRE is being evaluated in routine clinical practice at Imperial College Healthcare NHS Trust and Chelsea and Westminster Hospital.

In the context of implementing AI-based models in clinical practice, increasing attention is being paid to the interpretability and transparency of algorithmic decision-making. Processes, techniques, and methods that enable understanding and interpretation of the results and decisions generated by AI algorithms are referred to as explainable AI (XAI) [40]. Thoroughly tracing the process behind an algorithm’s output is important for patient safety, medical accountability, and the ethical acceptability of clinical decisions. XAI methods may help clinicians interpret which input features contributed most to a model’s output [41,42]. Moreover, clinicians can assess which factors, such as LDL-C levels, age, or elevated blood pressure, had the greatest influence on the predicted risk for an individual patient. This may facilitate interpretation and could increase clinician trust, although explanation quality varies between methods and models.

In the study by Rezk et al., the authors combined GBM algorithms with XAI to develop an explainable heart disease classification model [28]. In this study, this approach achieved high predictive performance, correctly classifying approximately 88–90% of cases and outperforming standalone classification models. By applying XAI, the authors identified features that contributed most strongly to model outputs, including patient age, blood pressure, LDL levels, and BMI. Additionally, it was possible to provide a detailed explanation of predictions for individual patients, highlighting which features had the greatest impact on the model’s decision. The use of XAI improved model transparency, enabling better understanding of its decisions at both the population and individual levels.

In lipid-based cardiovascular risk prediction models, XAI methods such as SHAP and LIME can further identify the contribution of individual lipid-related features to the final prediction. This may enable clinicians to determine whether LDL-C, lipoprotein(a), remnant cholesterol, or lipid variability are the primary drivers of cardiovascular risk in a given patient, thereby supporting more personalized lipid-lowering therapy and preventive strategies [4,18,28,40,41,42].

### Current Limitations and Future Perspectives

The limitations of AI-based cardiovascular models largely depend on their intended clinical use. The models discussed in this review include lipid phenotype classification, cardiovascular event prediction, mortality prediction, patient trajectory modeling, and absolute risk estimation, and therefore should not be evaluated as equivalent clinical tools. For models intended to predict cardiovascular events or estimate absolute risk, improved discrimination in retrospective datasets does not necessarily imply superiority over established guideline-based risk scores or readiness for clinical implementation. Such models should demonstrate incremental value in external validation cohorts, including appropriate calibration, reclassification metrics, decision-curve analysis, and prospective clinical impact studies. For other applications, such as lipid phenotype classification or patient trajectory modeling, the key question is not whether the model replaces traditional risk calculators, but whether it provides clinically actionable information that can improve decision-making. Most published models still require further validation before routine use, particularly because many were developed in retrospective datasets with heterogeneous endpoints, selected populations, and limited assessment of clinical utility [17,18,19,20,21,22,23,24,25,26,27,30,38,39]. Additional challenges include ensuring high-quality and standardized data [30,33], minimizing algorithmic bias, improving model interpretability to support clinician trust through XAI approaches [28,40,41,42], meeting regulatory requirements [39], and integrating AI tools into existing clinical workflows [41]. Addressing these challenges will be essential before AI-based cardiovascular models can be widely adopted in routine clinical practice.

The future clinical value of AI-based cardiovascular models may additionally depend on system-level implementation. In this regard, the Twin Transformation framework, combining digital innovation with more sustainable and efficient healthcare delivery, may provide a useful perspective for the integration of AI into cardiovascular care [43]. In this context, AI-based cardiovascular models may contribute not only to individualized risk stratification, but also to the redesign of cardiovascular care pathways. By integrating predictive analytics with digital health infrastructure, AI-supported approaches may facilitate more dynamic follow-up, earlier identification of patients requiring therapeutic intensification and more efficient use of healthcare resources. Nevertheless, the value of such transformation will most probably depend on robust validation, data interoperability, regulatory oversight, cost-effectiveness, and evidence that AI-supported workflows improve clinical decision-making and patient outcomes.

The clinical relevance of lipid profile-based AI models may also ultimately depend not only on risk stratification, but also on their ability to support more personalized dyslipidemia management. Recent publications on emerging technologies in dyslipidemia treatment have highlighted the potential integration of AI with nanotechnology-based therapeutic approaches as a future extension of lipid-centered precision medicine [44,45]. AI-based models could help identify lipid phenotypes, residual-risk patterns, or biomarker profiles associated with a higher likelihood of benefit from specific lipid-lowering strategies, while nanotechnology-based platforms may contribute to more targeted drug delivery or improved therapeutic efficiency. Thus, these approaches may complement AI-based cardiovascular event prediction by linking risk assessment with treatment personalization. However, their clinical implementation remains dependent on further evidence regarding safety, efficacy, validation in clinically relevant populations, cost-effectiveness, and long-term cardiovascular outcomes.

Moreover, future AI-based cardiovascular models may extend lipid-focused risk assessment by incorporating selected non-traditional biomarkers that reflect metabolic and inflammatory risk. One example is serum uric acid, which has been associated with hypertension, coronary artery disease, heart failure, inflammation, and oxidative stress. Future studies should determine whether adding serum uric acid and other non-traditional biomarkers to established lipid and clinical variables provides incremental predictive value and improves calibration, risk reclassification, and clinical utility in independently validated cohorts [46].

## 7. Conclusions

Artificial intelligence has the potential to improve cardiovascular risk prediction by enabling the analysis of complex relationships that cannot be captured by conventional risk calculators. Current evidence suggests that AI models using lipid profile parameters may help identify lipid phenotypes or lipid-related patterns associated with increased cardiovascular risk, while multimodal models integrating clinical, laboratory, imaging, longitudinal, and electronic health record data may further support cardiovascular event prediction, mortality prediction, or absolute risk estimation. However, these modeling tasks should not be interpreted as equivalent, and their clinical relevance depends on the population studied, outcome definition, validation strategy, and model calibration.

Despite these promising findings, reported improvements in predictive performance should be interpreted with caution. Many AI-based cardiovascular risk prediction models have been developed and evaluated retrospectively, often using heterogeneous datasets, different outcome definitions, and variable validation strategies. Consequently, apparent superiority over traditional risk scores may not necessarily translate into improved clinical decision-making. Most available models still have limited external validation and insufficient evidence demonstrating their clinical utility in real-world settings. Before widespread implementation, prospective multicenter studies and impact analyses are needed to compare lipid-only and multimodal models, establish standardized validation frameworks, and determine whether AI-assisted risk prediction improves clinical decision-making and patient outcomes. Furthermore, the adoption of XAI may be important for improving model transparency, supporting clinician trust, and facilitating safe integration into routine cardiovascular practice.

## Figures and Tables

**Table 1:** Summary of studies discussed in the review, including lipid-focused, multimodal, and broader cardiovascular applications. The table summarizes the study population, sample size, input variables, AI methods, modeling task, reported performance, external validation, and principal limitations of the included studies. The “model scope” indicates whether lipid-related variables constituted the primary model input, were incorporated as part of a broader multimodal dataset, or were not central to the model and the study was included as a contextual example of cardiovascular AI. Because the studies differ substantially in population characteristics, endpoints, prediction horizons, data sources, and validation strategies, their reported performance metrics should not be interpreted as directly comparable.

Study	Model Scope	Study Population	Sample Size	Input Variables	AI Method	Modeling Task	Performance Metrics Reported	External Validation/Calibration	Main Limitations
Xue et al., 2021 [17]	Lipid only	patients with ST-segment elevation myocardial infarction undergoing percutaneous coronary intervention	1355	TC, TG, HDL-C, LDL-C, apoA1, apoB, Lp(a)	Consensus clustering	Classification of lipid phenotypes and association with long-term clinical outcomes, including mortality and rehospitalization	Not reported; lipid phenogroups were compared for mortality and rehospitalization outcomes using adjusted hazard ratios	No	Single-center STEMI cohort; no longitudinal lipid measurements; relatively short follow-up
Du et al., 2024 [18]	Lipid focused	patients with hyperlipidemia followed for cardiovascular events over 3 years	23,548	Demographics; hypertension and diabetes history/duration; comorbidities; longitudinal blood pressure, lipid, glucose and BMI measures; lipid variability; remnant cholesterol; medication use; healthcare utilization	LightGBM, XGBoost, random forest, and SVM	Three-year cardiovascular event prediction in patients with hyperlipidemia	Best model (LightGBM): AUROC 0.883; AUPRC 0.931; accuracy 0.800; F1-score 0.850	No	Retrospective regional EHR cohort; no external validation or calibration assessment
Sadiq et al., 2025 [19]	Lipid only	pregnant women aged 15–49 years in Nigeria	112	TC, TG, HDL-C, LDL-C, and VLDL-C	Random Forest regression, boosting regression, decision tree regression	Prediction of dyslipidemia-associated cardiovascular disease risk in pregnancy	Best model (random forest): MSE 0.071, RMSE 0.266, R^2^ 0.95	No	Small single-center cross-sectional cohort; no external validation; no observed cardiovascular outcomes
Islam et al., 2024 [20]	Multimodal including lipids	UK Biobank-based study using baseline data and later imaging visit data	Over 500,000	Age, sex, Townsend Deprivation Index, blood pressure, ECG and echocardiographic variables, heart rate, cardiac output/index, body surface area, and physician-reported cardiovascular conditions	Linear SVM, radial basis function SVM, Gaussian process, decision tree, RF, neural network, AdaBoost, naive Bayes, and quadratic discriminant analysis	Classification of cardiovascular disease	Best accuracy 0.96; weighted F1-score 0.96; AUROC 0.96	No	Preprint, Single UK Biobank dataset; no external validation
Bagheri et al., 2020 [21]	Multimodal including lipids	Patients with manifest vascular disease or high cardiovascular risk	5603	Chest X-ray radiology reports; age, sex, smoking, systolic blood pressure, diabetes, HDL-C, total cholesterol, renal function, vascular disease history, and time since first CVD diagnosis	Multimodal BiLSTM	Cardiovascular risk prediction using longitudinal, multimodal EHR data	AUC 0.847; F1-score 83.8%; misclassification rate 14.3%	No	Preprint; complex multimodal EHR approach
Kwon et al., 2019 [22]	Multimodal including lipids	Patients with acute myocardial infarction from the Korean Working Group of Myocardial Infarction registry	22,875	Age, sex, BMI, pre-hospital cardiac arrest, systolic blood pressure, heart rate, Killip class, CK-MB, glucose, CRP, creatinine, LDL-C, and ST-segment elevation	Multilayer perceptron	In-hospital and 12-month mortality prediction after AMI	AUROC 0.905 for STEMI and 0.870 for NSTEMI	Yes; 10,723 patients from 23 hospitals not used for model development	validation limited to the same national registry; uncertain generalizability outside Korean AMI populations
Ward et al., 2020 [23]	Multimodal including lipids	adults from Northern California without prior CVD and not on statins at baseline	262,923	Age, sex, race, total cholesterol, HDL-C, systolic blood pressure, antihypertensive treatment, smoking, diabetes, plus diagnoses, medications, laboratory results, family history, socioeconomic factors	L2-regularized logistic regression, lasso logistic regression, random forest, gradient boosting machine, and extreme gradient boosting	Five-year ASCVD risk prediction	Best model (gradient boosting machine): AUROC 0.835	No	Single regional healthcare system; no external validation
Rao et al., 2025 [24]	multimodal and not lipid-focused	adults in England aged 25–84 years	3,000,000	Longitudinal diagnoses, age, medications, laboratory data including lipids	Transformer-based deep learning	Ten-year cardiovascular disease risk prediction	C-index 0.910 (95% CI 0.906–0.913)	Yes; held-out cohort from 98 independent general practices in England	Data from only one country; potential bias from baseline statin exclusion and treatment initiation during follow-up
Rao et al., 2026 [25]	multimodal and not lipid-focused	Heart failure patients in England	403,534	Multimorbidity patterns, longitudinal clinical records and time-dependent clinical changes	Transformer-based deep learning	All-cause mortality prediction in heart failure	C-index 0.845 in UK validation and 0.802 in USA validation	Yes; practice-level UK validation and independent MIMIC-IV cohort from the USA	Heart-failure-specific; mortality endpoint rather than primary CVR prediction
Renc et al., 2024 [26]	not lipid-focused and not CVD-specific	Hospitalized and critically ill patients from the MIMIC-IV database	Over 200,000	demographics, admissions and ICU stays, diagnoses, procedures, medicatio	Decoder-only transformer	Prediction of future health trajectories	Hospital mortality AUROC 0.921; ICU mortality AUROC 0.927; ICU readmission AUROC 0.807; 30-day hospital readmission AUROC 0.749	No	Single-center acute-care dataset; no external or prospective validation
Sau et al., 2024 [27]	non-lipid implementation example	Secondary-care patients	189,539 patients (over 1 million ECGs)	Raw waveform data from a single 12-lead ECG	Residual convolutional neural network	Prediction of mortality and future cardiovascular outcomes, including myocardial infarction, heart failure and arrhythmias	C-index: all-cause mortality 0.775; ventricular arrhythmia 0.760; ASCVD 0.696; heart failure 0.787; external mortality cohorts 0.638–0.773	Yes; five cohorts from the USA, Brazil, and the United Kingdom	Retrospective design; heterogeneous external cohorts and variable performance
Rezk et al., 2024 [28]	non lipid implementation example	Patients from a Kaggle heart disease dataset, including individuals with and without coronary heart disease	1190	Age, sex, chest pain type, resting blood pressure, serum cholesterol, fasting blood glucose, resting ECG, maximum heart rate, exercise-induced angina, ST depression, and ST-segment slope	XGBoost—LightBoost with SHAP and LIME explanations	Heart disease classification with explainability	Test accuracy 96.22%; recall 95.8%; precision 96.4%; AUC 95.75%	No	Single public dataset with unclear clinical provenance

## Data Availability

No new data were created or analyzed in this study. Data sharing is not applicable to this article.
